# Structure and Function of Intra–Annual Density Fluctuations: Mind the Gaps

**DOI:** 10.3389/fpls.2016.00595

**Published:** 2016-05-06

**Authors:** Giovanna Battipaglia, Filipe Campelo, Joana Vieira, Michael Grabner, Veronica De Micco, Cristina Nabais, Paolo Cherubini, Marco Carrer, Achim Bräuning, Katarina Čufar, Alfredo Di Filippo, Ignacio García-González, Marcin Koprowski, Marcin Klisz, Alexander V. Kirdyanov, Nikolay Zafirov, Martin de Luis

**Affiliations:** ^1^Department of Environmental, Biological and Pharmaceutical Sciences and Technologies, Second University of NaplesCaserta, Italy; ^2^Centre for Bio-Archaeology and Ecology, PALECO Ecole Pratique des Hautes Etudes, Institut des Sciences de l'Evolution, University of Montpellier 2Montpellier, France; ^3^Euro-Mediterranean Center on Climate ChangeLecce, Italy; ^4^Department of Life Sciences, Centre for Functional Ecology, University of CoimbraCoimbra, Portugal; ^5^Institute of Wood Technology and Renewable Resources, University of Natural Resources and Life SciencesVienna, Austria; ^6^Department of Agricultural Sciences, University of Naples Federico IINaples, Italy; ^7^Swiss Federal Research Institute WSLBirmensdorf, Switzerland; ^8^Department of Land, Environment, Agriculture and Forestry, University of PaduaPadua, Italy; ^9^Department of Geography and Geosciences, Institute of Geography, Friedrich-Alexander-University Erlangen-NurembergErlangen, Germany; ^10^Department of Wood Science and Technology, Biotechnical Faculty, University of LjubljanaLjubljana, Slovenia; ^11^Department Agricultural and Forestry, Università degli Studi della TusciaViterbo, Italy; ^12^Departamento de Botánica, Escuela Politécnica Superior, University of Santiago de CompostelaLugo, Spain; ^13^Faculty of Biology and Environment Protection, Nicolaus Copernicus UniversityToruñ, Poland; ^14^Department of Silviculture and Genetics, Forest Research Institute in PolandBudynek, Poland; ^15^V.N. Sukachev Institure of Forest SB RASKrasnoyarsk, Russia; ^16^Department of Math Methods and IT, Siberian Federal UniversityKrasnoyarsk, Russia; ^17^Department of Plant Pathology and Chemistry, University of ForestrySofia, Bulgaria; ^18^Department of Geography and Regional Planning—IUCA, University of ZaragozaZaragoza, Spain

**Keywords:** IADF, tree-ring, wood anatomy, stable isotopes, network analysis, wood formation

## Abstract

Tree rings are natural archives of climate and environmental information with a yearly resolution. Indeed, wood anatomical, chemical, and other properties of tree rings are a synthesis of several intrinsic and external factors, and their interaction during tree growth. In particular, Intra-Annual Density Fluctuations (IADFs) can be considered as tree-ring anomalies that can be used to better understand tree growth and to reconstruct past climate conditions with intra-annual resolution. However, the ecophysiological processes behind IADF formation, as well as their functional impact, remain unclear. Are IADFs resulting from a prompt adjustment to fluctuations in environmental conditions to avoid stressful conditions and/or to take advantage from favorable conditions? In this paper we discuss: (1) the influence of climatic factors on the formation of IADFs; (2) the occurrence of IADFs in different species and environments; (3) the potential of new approaches to study IADFs and identify their triggering factors. Our final aim is to underscore the advantages offered by network analyses of data and the importance of high-resolution measurements to gain insight into IADFs formation processes and their relations with climatic conditions, including extreme weather events.

## IADF formation and position

Intra-Annual Density Fluctuations (IADFs) are variations in wood density that are defined by the presence of earlywood-like cells within latewood and (or) by the presence of latewood-like cells within earlywood (Fritts, 2001). Such anatomical structures may hamper cross-dating and any further analyses of tree-ring series (Cherubini et al., 2003). Thus, IADFs have long been considered by dendrochronologists as the “Ugly Duckling” of wood anatomical features, and species forming them have often been discarded for climate reconstructions (Lorimer et al., 1999) and used as indicators of particular events such as flood-regime or air pollution (see Wimmer, 2002 for review). During the 2000s, the “Ugly Duckling” turned into a “Beautiful Swan,” when different studies demonstrated the potential of these anatomical features for ecological, environmental, and climatological interpretations (Wimmer et al., 2000; Rigling et al., 2001; De Micco et al., 2007; Campelo et al., 2007a,b; De Luis et al., 2007). Since then, the importance of IADFs has been widely recognized and the number of papers dealing with them has increased significantly (De Micco et al., 2016). It has been underlined that IADFs may provide accurate information at the seasonal level (Glock and Agerter, 1960; Tessier et al., 1997; Bräuning, 1999; Campelo et al., 2007b; Battipaglia et al., 2010, 2014; De Micco et al., 2012), allowing a more detailed climate analysis within the growing season (Wimmer et al., 2000; Novak et al., 2013a,b, 2016).

Variations in IADF features can be used to reconstruct past environmental conditions, and IADF relative position within the ring can be used to estimate when a specific environmental factor occurred (Figure S1). Campelo et al. (2007b) classified a band of latewood-like cells at the end of earlywood in *Pinus pinea* as an “IADF type E^+^,” corresponding to a gradual transition from early- to latewood (Figure S1). They suggested that those IADFs could be linked to soil water conditions during late spring, hypothesizing that rainfall events in late spring could delay the transition from early- to latewood. Later in the growing season the cambium can reverse latewood production forming again earlywood-like cells. When a band of latewood-like cells is located within earlywood the IADF is labeled as “type E” (Campelo et al., 2007b). This type of IADF (Figure S1) seems to be uncommon in Mediterranean pine species (Vieira et al., 2010; Rozas et al., 2011; Campelo et al., 2013) probably because regular weather conditions during spring can assure continuous growth (Olano et al., 2012), or trees are able to minimize episodic events of water stress during the early growing season (Loustau et al., 1996; Borghetti et al., 1998). In contrast, the formation of IADF type E was found to be rather frequent in other environments: for example, in *Pinus nigra* sampled in the Vienna Basin, this type of IADF was triggered by a combination of wet April, dry May, and wet June and it was related to the water-table level (Wimmer et al., 2000). In *Erica arborea* and *Arbutus unedo*, hardwood species growing in the Mediterranean basin, this type of IADF was also frequent and triggered by summer drought conditions (Battipaglia et al., 2010, 2014; De Micco et al., under revision). In hardwood species the comparison of series of vessel lumen size between tree rings with and without IADFs suggested that: (a) IADF position is related to the period of the season when stressful conditions priming the fluctuation occur, (b) the width of the IADF indicates the duration of conditions triggering its formation (Campelo et al., 2007a; De Micco et al., 2014). Most studies dealing with IADFs found that their frequency increased close to the end of the tree ring (Rigling et al., 2001; Rozas et al., 2011). Two types of latewood IADFs have been classified considering the position within latewood: the first type is characterized by earlywood-like cells within latewood (IADF L; Figure S1) and the other located between latewood and earlywood of the next ring and characterized by intermediate anatomical traits (IADF L^+^; Figure S1) (Campelo et al., 2007b). In both cases, they were mainly associated with favorable conditions occurring after the summer drought, in early autumn (L) or in late autumn (L^+^) (Rigling et al., 2001; Masiokas and Villalba, 2004; Campelo et al., 2007b; Battipaglia et al., 2010, 2014). Although, this first classification could be criticized for the fact that IADFs E^+^ and L^+^ do not correspond to a true fluctuation in wood density, it is important to question the value of the position of IADFs as a proxy for past climate. By using the relative position of IADFs within tree rings it is possible to improve the temporal resolution of tree-ring series (De Micco et al., 2014), especially in areas where the growing season is long, such as in the Mediterranean region (Rozas et al., 2011; De Luis et al., 2011a).

The identification of the environmental conditions triggering IADF formation is based on linear correlations between climatic variables and IADF chronologies, highlighting the importance of water conditions during the growing season in their formation. However, these correlations are not enough to fully understand the process behind IADF formation, namely at the level of cambial activity and cell differentiation processes. Are IADFs the result of cambial reactivation? Are IADF cells already present in the cambial zone undergoing differentiation? Are latewood IADFs caused by changes in the cell enlargement and/or cell wall deposition phase? These are fundamental questions that can only be answered by monitoring xylogenesis at a weekly time scale, and relating it to intra-ring variations of cell features. Studies on cambial dynamics and wood production can help us to understand the physiological mechanisms behind IADFs formation (Camarero et al., 2010; Vieira et al., 2015). There have been recently major developments in this field, leading to a detailed description of the timings of cambial activity, duration of cell production and differentiation phases and response of cambium to environmental conditions in different species and environments (De Luis et al., 2007; Camarero et al., 2010; Cuny et al., 2014). These studies showed that cambial activity in the Mediterranean region presents a high year-to-year variability, strongly dependent on climate (Vieira et al., 2014a). Cambial activity in the Mediterranean, as in other temperate environments, starts in spring in response to warm temperatures and increasing photoperiod (Vieira et al., 2014b), with periclinal cell divisions of the vascular cambium and the production of earlywood tracheids. It reaches a maximum around May and then, when water becomes less abundant, cambial activity slowly decreases, reaching a minimum in the summer months (Camarero et al., 2010), when latewood tracheids are produced (Uggla et al., 2001). Water availability is fundamental for cell division and turgor-driven cell expansion (Kutschera and Niklas, 2013). Expansion only starts once a threshold of turgor pressure is achieved and the pressure applied by the water-filled vacuole against the cell wall determines the tracheid final size (Oribe et al., 2003). The formation of the latewood cells is expected during summer, while the earlywood-like cells can be formed in autumn, if favorable climatic conditions return. Several studies under Mediterranean climatic conditions suggested that cambial activity could show a bimodal pattern with two main peaks: one in spring and the other in autumn (De Luis et al., 2011a,b; Battipaglia et al., 2014; Vieira et al., 2015). Since cambial reactivation after a dry summer is not always observed, a facultative bimodal pattern is the best way to describe cambial activity in Mediterranean environments. The tracheids differentiated after summer drought differ from those previously formed in latewood, since their cell wall thickness to lumen diameter ratio is lower than in true latewood (Carvalho et al., 2015; Vieira et al., 2015). Thus, tracheids forming IADFs L have larger radial cell and lumen dimensions than true latewood tracheids. Differentiating tracheids can expand beyond the usual radial diameter of latewood tracheids, if water is available. Indeed, the lumen area of a tracheid depends on turgor pressure and duration of cell enlargement (Cuny et al., 2014). These results suggest that the formation of latewood IADFs in the Mediterranean area are defined during the enlargement phase, whereas it is possible that latewood IADFs formed at higher altitudes and latitudes are caused by changes in the cell wall deposition phase. In colder environments, tracheid differentiation must be concluded before the onset of winter (Rossi et al., 2008) and IADF L^+^ can be formed if there is not enough time to complete the deposition phase due to a fast drop in air temperatures. However, formation and ontogenesis of this kind of IADFs are still under debate.

## Impact of IADFs on tree hydraulics

One important gap in IADF research is the functional role played by these anatomical structures on tree hydraulics (Wilkinson et al., 2015). It is known that the size of conduits (e.g., tracheids and vessels) is related to the hydraulic conductivity, while protection from drought-induced embolism is a function of the ability to prevent air-seeding and this is strongly related with the number and size of pits, thus indirectly with lumen size (Hacke et al., 2004; Pittermann et al., 2006). Earlywood IADFs, characterized by latewood-like cells within earlywood, potentially represent a fraction of the earlywood with a lower hydraulic conductivity, while the opposite occurs for IADFs located in latewood. Small increases in tracheid lumen can dramatically increase hydraulic conductivity because flow rate is proportional to the fourth power of the tracheid radius (Tyree and Ewers, 1991). Thus, it is important to quantify their impact on tree hydraulics, because currently we only have indirect observations (Campelo et al., 2007b). It can be assumed that all cells forming IADFs are conductive in order to quantify IADFs impact on the total hydraulic conductivity. Afterwards, a more experimental approach is needed to check if IADFs are functional from a hydraulic point of view. It is also important to characterize the cells forming the IADFs, namely their lumen diameter, length, number, and size of pits, as these anatomical characteristics will affect their hydraulic conductivity.

## Objective assessment of IADFs

Visual identification of IADFs in conifers is only possible through the analysis of variations in tracheid features (e.g., cell and lumen diameter, and cell-wall thickness). The accuracy of the visual macroscopic identification of IADFs depends on many parameters, such as the quality of wood surface polishing, microscope magnification and criteria used to distinguish IADFs. Since visual identification of IADFs is based on qualitative criteria rather than on quantitative measurements, the subjectivity of the operator can also be one of the major sources of error. Intra-ring variations in tracheid anatomy, and consequently IADFs, can also be identified through quantitative measurements of tracheid features or tracheidograms (Hetzer et al., 2014; Ziaco et al., 2014; Carvalho et al., 2015; Campelo et al., 2016) and image analysis of X-ray densitometry profiles (Cherubini et al., 2013; Gonzalez-Benecke et al., 2015; Wilkinson et al., 2015). Image analysis avoids the long and tedious procedure of visual examination of wood samples and IADF characteristics can be computed automatically (e.g., relative position within the tree ring and IADF-band width). Image analysis also precludes the operator's subjectivity and provides the size distribution of tracheid features (e.g., lumen diameter and cell-wall thickness). However, it is highly recommended that IADFs recognized automatically by algorithms to be compared with those obtained visually by an expert of wood anatomy for an initial calibration to guarantee the correctness of the criteria used for their identification. All general constraints listed for the identification of IADF in softwoods apply also to hardwoods, where IADF analysis is even more complicated due to the occurrence of different cell types and the spatial distribution of vessels, which are usually not arranged in regular rows like softwood tracheids. Further studies are needed because a number of different anatomical functional traits seem to work for IADF identification in hardwoods, but they appear to be species-specific (De Micco et al., 2015). Furthermore, the fact that many features can be involved (e.g., vessel size, fiber wall thickness, spatial arrangement of cells) opens the possibility to define new types of IADFs.

Within this framework, sharpening the focus at the tissue scale and analyzing various xylem histological traits seemingly represents one of the most promising approaches. Recent methodological advances in quantitative wood anatomy (Gärtner and Schweingruber, 2013; Von Arx and Carrer, 2014) allow efficient development of multi-centennial time series of xylem anatomical traits mostly related to the type and number of cells per ring and to cell-lumen and cell-wall dimensions. These advances not only improve the length of the generated time-series, but most of all the robustness of the measurements. With the currently available computer capacity combined with specific software, accurately tailored to analyze wood anatomical traits, it is possible to apply a more thorough and unbiased approach considering all cells within each wood anatomical image. This can outperform the previous practice of measuring xylem traits along a few selected radial cell files as it allows information to be collected on hundreds to several thousands of cells per ring (Figure S2). This, together with improved perception of the long-term ontogenetic change in xylem-cell dimension (Carrer et al., 2015), clearly opens the door for a sound statistical analysis not just of IADFs frequency but also on their extension, intensity, position within the ring, or on the relative role of different cell traits to classify different IADFs types that cannot be unambiguously distinguished by visual inspection.

## Stable isotopes approach

Quantitative wood anatomy has recently been coupled with stable isotope (δ^13^C and δ^18^O) measurements (De Micco et al., 2007; Vaganov et al., 2009; Battipaglia et al., 2010, 2014) to characterize IADFs, offering new perspectives in the interpretation of IADFs in relation to physiological and ecological processes. What is still unsolved is if the stable isotope signals can help us to identify the different types of IADFs within a ring. Battipaglia et al. (2010, 2014) demonstrated with *in continuum* stable isotope measurements in hardwood species that IADFs have a unique isotopic signature linked to their position, and are completely different from the correspondent well-known earlywood-latewood isotopic range values (Helle and Schleser, 2004; Vaganov et al., 2009). Here, we performed a preliminary study analyzing IADFs of different species at different sites (*P. pinea* from Italy, *Pinus halepensis* from Spain and Slovenia; *Pinus pinaster* from Portugal, *Larix decidua* from Poland, and *L. decidua x kaempferi* from Austria), in order to verify a possible common isotopic signal at intra-annual scale for each type of IADF. IADF L was found in all sites and species, whereas IADF L^+^ and E^+^ were only present in 66% of the sites and type E only in 50%. The carbon and oxygen isotopic signals of the different kinds of fluctuations were consistent between sites and species with differences between IADFs type E, E^+^, and L and none between L and L^+^, supporting the hypothesis that the L^+^ can be considered as a transitional wood and not as a true fluctuation (Table S1, Figures S3, S4). Although a more complete analysis is required in order to completely understand the link between isotope signals, position, and climatic parameters triggering IADF formation, stable isotopes seem to be a powerful tool not only to increase physiological information on plant responses to climate, but also for the objective identification of each IADF type.

## IADF occurrence and network approach

IADFs have been reported in several species (hardwoods and softwoods), and regions across a wide gradient of temperature and rainfall availability, from tropical to subarctic, to semi-arid and arid environments (De Micco et al., 2016). The majority of studies have been conducted in Mediterranean ecosystems where the highest frequency of IADFs has been reported, particularly in conifers such as *Pinus* spp., and where several efforts have been made to analyze the characteristics and ecological meaning of IADFs (Bräuning, 1999; Wimmer et al., 2000; Rigling et al., 2002; De Micco et al., 2007; Campelo et al., 2007b, 2013, 2015; De Luis et al., 2007, 2011a,b; Vieira et al., 2009, 2010; Camarero et al., 2010; Rozas et al., 2011; Nabais et al., 2014; Novak et al., 2013a,b). Under boreal or temperate climate, IADFs have been observed in 9% of the tree rings at maximum (Wimmer et al., 2000; Rigling et al., 2001; Copenheaver et al., 2006), while under Mediterranean climate, they have been observed in up to 15–32% of rings (Campelo et al., 2007b; Bogino and Bravo, 2009; Vieira et al., 2009; Novak et al., 2013a).

**Figure 1:**
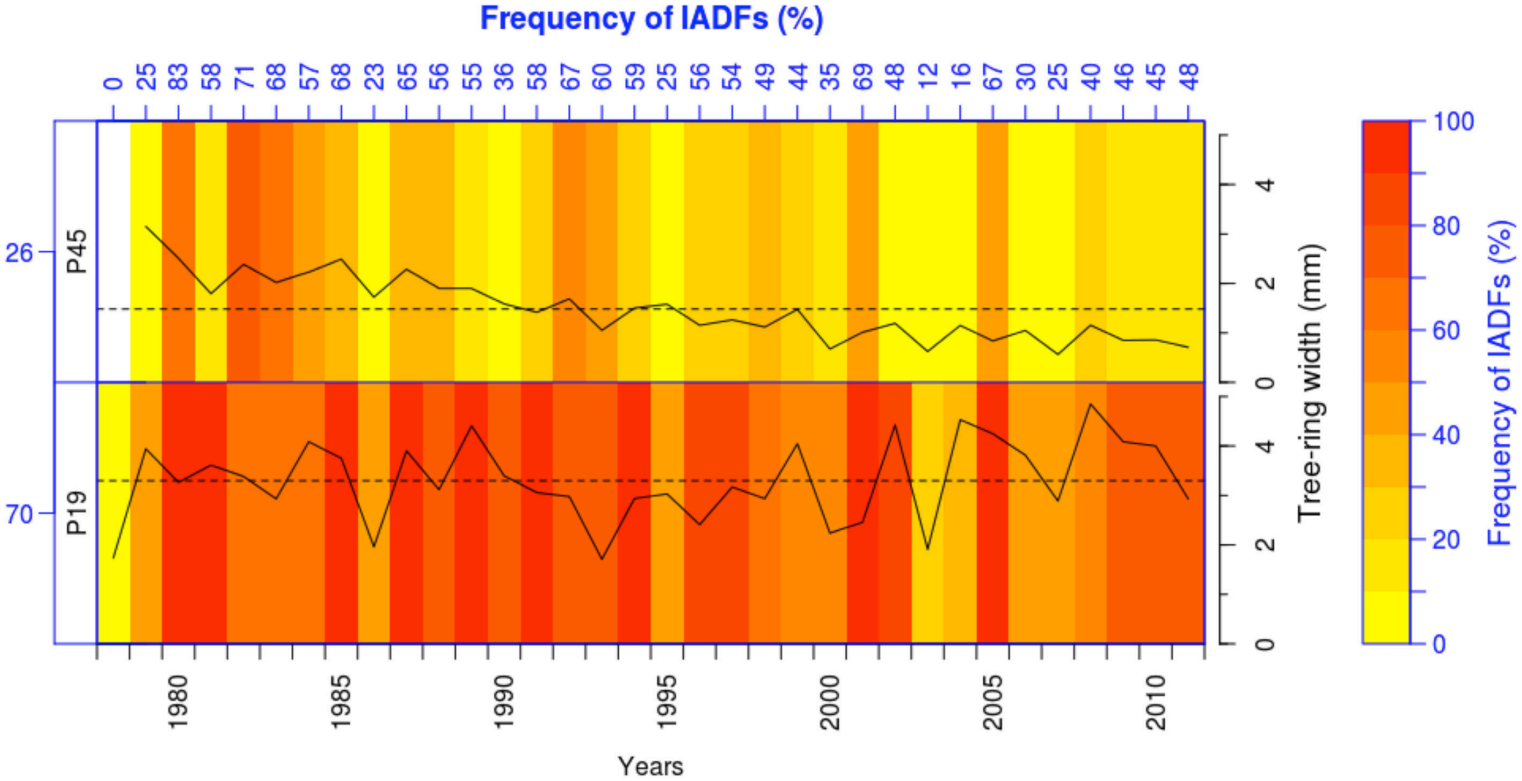
**Heatmap showing IADFs for two different provenances of *Abies alba*: a fast-growing (P19, *n* = 17) and a slow-growing provenance (P45, *n* = 24)**. The slow-growing provenance showed less IADFs (26%) than the fast-growing provenance (70%). *Solid lines* indicate the tree-ring width and *dashed lines* the mean tree-ring width; the mean tree-ring width in the fast-growing provenance (3.3 mm) was twice as wide than in the slow-growing provenance (1.5 mm).

The role of sex and genetics on the occurrence of IADFs has only recently been investigated. Olano et al. (2015), studying IADF frequency in *Juniperus thurifera* growing in two sites with contrasting hydrological conditions in Spain, reported that female trees present the highest frequency of IADFs reflecting their opportunistic water use strategy. Tree-breeding studies have shown the influence of provenance on tree growth and wood properties (Rozenberg et al., 2002; Hannrup et al., 2004; Klisz et al., under revision). For example, Rozenberg et al. (2002) found that different wood density parameters, including density fluctuations in earlywood and their position within the tree ring showed high heritability. However, the effect of provenances on IADF formation has not been investigated in detail yet, but we can expect that IADF frequency should differ in provenances with different growth rates. As experimental approach, we investigated the influence of provenance on IADF formation by comparing two contrasting provenances (in terms of tree growth) from a long-term trial (1970–2011) of *Abies alba* (George et al., 2015) grown in eastern Austria. The mean tree-ring width of the fast-growing provenance (Slovakia, P19; 3.3 mm) was twice as wide than the slow-growing provenance (Italy, P45; 1.5 mm). As expected the highest IADF frequency was observed in the fast-growing provenance (Figure 1), highlighting the necessity for further investigation of the genetic influence on IADF occurrence.

**Figure 2:**
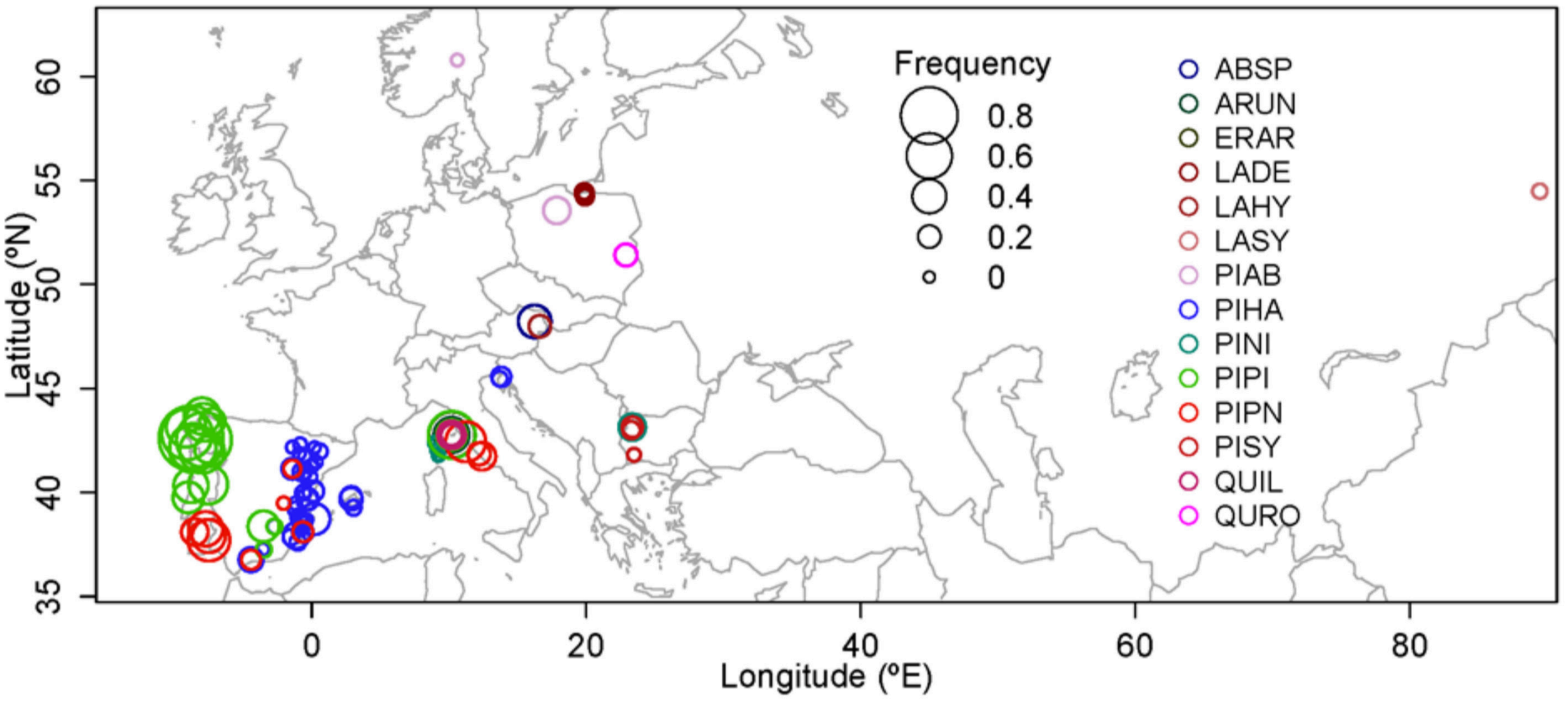
**IADF frequency as observed in the 108 populations included in the catalog created during COST FP1106 “STReESS.”** ABSP, *Abies* species; ARUN, *Arbutus unedo* L.; ERAR, *Erica arborea* L.; LADE, *Larix decidua* Mill.; LAHY, *Larix decidua x kaempferi*; LASY, *Larix sibirica* Ledeb; PIAB, *Picea abies* (L.) Karst; PIHA, *Pinus halepensis* Mill.; PINI, *Pinus nigra* Arn.; PIPI, *Pinus pinaster* Aiton; PIPN, *Pinus pinea* L.; PISY, *Pinus sylvestris* L.; QUIL, *Quercus ilex* L.; QURO, *Quercus robur* L.

The consistency of the climatic signal among different pine species and areas suggested that a large-scale network of IADFs in the Mediterranean region could help to study intra-annual climate variability (Zalloni et al., 2016). In the framework of the FPS COST Action FP1106 STReESS (Studying Tree Responses to Extreme Events: a SynthesiS), a catalog and database of IADF occurrence and anatomical and isotopic features have been developed, consisting of data collected on different species across a large geographical range. This unique and novel catalog includes IADF identification and measurements in 10 countries, 14 species, and 108 tree populations with a total of 2199 trees (3670 cores) and 234,262 tree rings. In this perspective we present a first exploratory analysis on IADFs showing a wide range of variability in IADF frequency (Figure 2, Table S2), with sites where IADFs are nearly absent (minimum frequency of 0.9% in high-elevation *P. nigra* on Corsica) and others where IADFs are present in nearly all tree rings (maximum of 93% in *P. pinaster* in Galicia, Spain). The network approach offers important advantages since it overcomes limitations due to tree-age and tree-size effects (Vieira et al., 2009; Novak et al., 2013a; Campelo et al., 2015) and to local replication (Zalloni et al., 2016). It also provides a unique possibility to interpret the relationship between IADF frequency and the main climate factors promoting their formation at a regional scale as described by Zalloni et al. (2016) for *P. halepensis, P. pinea*, and *P. pinaster* across their distribution range.

## Conclusions

To maximize the extraction of environmental information from IADFs, more researches on IADF formation and data about IADF frequency are needed. There is also a need to classify IADFs more precisely, and to quantify their wood anatomical features. In this context, a network approach could help to identify not only the main climatic drivers of IADF formation, but also to clarify the functional role of IADFs across different environments and species. The catalog presented here will be further explored and new data will be welcome from different environments and species aiming to create a unique network between scientists working with IADFs. This would help us to answer the large number of open questions and to fill the current gaps on IADFs research.

Further, we believe that one urgent issue still under debate is the identification of IADFs using wood quantitative approaches. Until now, each operator has used his own ability (that depends on experience) to recognize IADFs and to assign them to earlywood or latewood. In many tree species, the correct identification of IADFs is more difficult because the transition between earlywood and latewood is not straightforward and unequivocal. Given the subjective nature of IADF identification, the operator must be well trained and experienced. However, an intrinsic error due to the operator's subjectivity will always remain during the process of IADF identification. To overcome this drawback, machine learning based approaches should be specifically developed to recognize IADFs.

## Author contributions

GB and MD gave a substantial contribution to the conception and design of the study. GB, MD, AB, AK, AD, CN, KC, MKl, MK, MG, NZ, IG contributed to the supply of data for the network. MD was in charge of network analyses. GB performed stable isotopes analyses. GB wrote the first draft of the manuscript. MD, FC, JV, VD, CN, MG, MC, PC contributed to writing specific sections of the manuscript. All authors contributed to manuscript revision, read, and approved the submitted manuscript.

### Conflict of interest statement

The authors declare that the research was conducted in the absence of any commercial or financial relationships that could be construed as a potential conflict of interest.

## References

[B1] Battipaglia G. De Micco V. Brand W. A. Linke P. Aronne G. Saurer M.. (2010). Variations of vessel diameter and δ^13^C in false rings of *Arbutus unedo* L. reflect different environmental conditions. New Phytol. 188, 1099–1112. 10.1111/j.1469-8137.2010.03443.x 20840507

[B2] Battipaglia G. De Micco V. Brand W. A. Saurer M. Aronne G. Linke P.. (2014). Drought impact on water use efficiency and intra-annual density fluctuations in *Erica arborea* on Elba (Italy). Plant Cell Environ. 37, 382–391. 10.1111/pce.12160 23848555

[B3] Bogino S. Bravo F. (2009). Climate and intraannual density fluctuations in *Pinus pinaster* subsp. mesogeensis in Spanish woodlands. Can. J. For. Res. 39, 1557–1565. 10.1139/X09-074

[B4] Borghetti M. Cinnirella S. Magnani F. Saracino A. (1998). Impact of long-term drought on xylem embolism and growth in *Pinus halepensis* Mill. Trees 12, 187–195. 10.1007/pl00009709

[B5] Bräuning A. (1999). Dendroclimatological potential of drought-sensitive tree stands in southern Tibet for the reconstruction of monsoonal activity. IAWA J. 20, 325–338. 10.1163/22941932-90000695

[B6] Camarero J. J. Olano J. M. Parras A. (2010). Plastic bimodal xylogenesis in conifers from continental Mediterranean climates. New Phytol. 185, 471–480. 10.1111/j.1469-8137.2009.03073.x 19895415

[B7] Campelo F. Gutiérrez E. Ribas M. Nabais C. Freitas H. (2007a). Relationships between climate and double rings in *Quercus ilex* from northeast Spain. Can. J. For. Res. 37, 1915–1923. 10.1139/X07-050

[B8] Campelo F. Nabais C. Carvalho A. Vieira J. (2016). tracheideR – an R package to standardize tracheidograms. Dendrochronologia 37, 64–68. 10.1016/j.dendro.2015.12.006

[B9] Campelo F. Nabais C. Freitas H. Gutiérrez E. (2007b). Climatic significance of tree-ring width and intra-annual density fluctuations in *Pinus pinea* from a dry Mediterranean area in Portugal. Ann. For. Sci. 64, 229–238. 10.1051/forest:2006107

[B10] Campelo F. Vieira J. Nabais C. (2013). Tree-ring growth and intra-annual density fluctuations of *Pinus pinaster* responses to climate: does size matter? Trees 27, 763–772. 10.1007/s00468-012-0831-3

[B11] Campelo F. Vieira J. Battipaglia G. de Luis M. Nabais C. Freitas H.. (2015). Which matters most for the formation of intra-annual density fluctuations in *Pinus pinaster*: age or size? Trees 29, 237–245. 10.1007/s00468-014-1108-927132263

[B12] Carrer M. Von Arx G. Castagneri D. Petit G. (2015). Distilling allometric and environmental information from time series of conduit size: the standardization issue and its relationship to tree hydraulic architecture. Tree Physiol. 35, 27–33. 10.1093/treephys/tpu108 25576756

[B13] Carvalho A. Nabais C. Vieira J. Rossi S. Campelo F. (2015). Plastic response of tracheids in *Pinus pinaster* in a water-limited environment: adjusting lumen size instead of wall thickness. PLoS ONE 10:e0136305. 10.1371/journal.pone.0136305 26305893 PMC4549277

[B14] Cherubini P. Gartner B. L. Tognetti R. Bräker O. U. Schoch W. Innes J. L. (2003). Identification, measurement and interpretation of tree rings in woody species from Mediterranean climates. Biol. Rev. 78, 119–148. 10.1017/S1464793102006000 12620063

[B15] Cherubini P. Humbel T. Beeckman H. Gäertner H. Mannes D. Pearson C.. (2013). Olive tree-ring problematic dating: a comparative analysis on Santorini (Greece). PLoS ONE 8:e54730. 10.1371/journal.pone.0054730 23382949 PMC3557290

[B16] Copenheaver C. A. Pokorski E. A. Currie J. E. Abrams M. D. (2006). Causation of false ring formation in *Pinus banksiana*: a comparison of age, canopy class, climate, and growth rate. For. Ecol. Manag. 236, 348–355. 10.1016/j.foreco.2006.09.020

[B17] Cuny H. E. Rathgeber C. B. K. Frank D. Fonti P. Fournier M. (2014). Kinetics of tracheid development explain conifer tree-ring structure. New Phytol. 203, 1231–1241. 10.1111/nph.12871 24890661

[B18] De Luis M. Gricar J. Cufar K. Raventós J. (2007). Seasonal dynamics of wood formation: a comparison between pinning, microcoring and dendrometer measurements. IAWA J. 28, 389–404. 10.1007/s10342-007-0199-x

[B19] De Luis M. Novak K. Raventós J. Gricar J. Prislan P. Cufar K. (2011a). Climate factors promoting intra-annual density fluctuations in Aleppo pine (*Pinus halepensis*) from semiarid sites. Dendrochronologia 29, 163–169. 10.1016/j.dendro.2011.01.005

[B20] De Luis M. Novak K. Raventós J. Gricar J. Prislan P. Cufar K. (2011b). Cambial activity, wood formation and sapling survival of *Pinus halepensis* exposed to different irrigation regimes. For. Ecol. Manage. 262, 1630–1638. 10.1016/j.foreco.2011.07.013

[B22] De Micco V. Battipaglia G. Balzano A. Cherubini P. Aronne G. (2015). Are wood fibres as sensitive to environmental conditions as vessels in tree rings with intra-annual density fluctuations (IADFs) in Mediterranean species? Trees. 1–13. 10.1007/s00468-015-1338-5

[B23] De Micco V. Battipaglia G. Brand W. A. Linke P. Saurer M. Aronne G. (2012). Discrete versus continuous analysis of anatomical and δ^13^C variability in tree rings with intra annual density fluctuations. Trees 26, 513–524. 10.1007/s00468-011-0612-4

[B24] De Micco V. Battipaglia G. Cherubini P. Aronne G. (2014). Comparing methods to analyse anatomical features of tree rings with and without intra-annual density fluctuations (IADFs). Dendrochronologia 32, 1–6. 10.1016/j.dendro.2013.06.001

[B25] De Micco V. Campelo F. de Luis M. Bräuning A. Grabner M. Battipaglia G. (2016). Formation of Intra-Annual-Density-Fluctuations in tree rings: how, when, where and why? IAWA J. 37, 232–259. 10.1163/22941932-20160132

[B26] De Micco V. Saurer M. Aronne G. Tognetti R. Cherubini P. (2007). Variations of wood anatomy and δ^13^C within-tree rings of coastal *Pinus pinaster* showing intra-annual density fluctuations. IAWA J. 28, 61–74. 10.1163/22941932-90001619

[B27] Fritts H. C. (2001). Tree Rings and Climate. London: The Blackburn Press.

[B28] Gärtner H. Schweingruber F. H. (2013). Microscopic Preparation Techniques for Plant Stem Analysis. Remagen: Verlag Dr. Kessel.

[B29] George J. P. Schueler S. Karanitsch-Ackerl S. Mayer K. Klumpp R. T. Grabner M. (2015). Inter- and intra-specific variation in drought sensitivity in *Abies spec*. and its relation to wood density and growth traits. Agr. For. Meteorol. 214–215, 430–443. 10.1016/j.agrformet.2015.08.268 PMC5049588 27713591

[B30] Glock W. S. Agerter S. R. (1960). Classification and Multiplicity of Growth Layers in the Branches of Trees: At the Extreme Lower Forest Border. Washington, DC: Smithsonian Institution.

[B31] Gonzalez-Benecke C. A. Riveros-Walker A. J. Martin T. A. Peter G. F. (2015). Automated quantification of intra-annual density fluctuations using microdensity profiles of mature *Pinus taeda* in a replicated irrigation experiment. Trees 29, 185–197. 10.1007/s00468-014-1103-1

[B32] Hacke U. G. Sperry J. S. Pittermann J. (2004). Analysis of circular bordered pit function II. Gymnosperm tracheids with torus-margo pit membranes. Am. J. Bot. 91, 386–400. 10.3732/ajb.91.3.386 21653394

[B33] Hannrup B. Cahalan C. Chantre C. Grabner M. Karlsson B. Le Bayon I. (2004). Genetic parameters of growth and wood quality traits in *Picea abies*. Scand. J. For. Res. 19, 14–29 10.1080/02827580310019536

[B34] Helle G. Schleser H. (2004). Beyond CO_2_-fixation by Rubisco- an interpretation of ^13^C / ^12^C variations in tree rings from novel intraseasonal studies on broad-leaf trees. Plant Cell Environ. 27, 367–380 10.1111/j.0016-8025.2003.01159.x

[B35] Hetzer T. Bräuning A. Leuschner H.-H. (2014). High-resolution climatic analysis of wood anatomical features in Corsican pine from Corsica (France) using latewood tracheid profiles. Trees 28, 1279–1288. 10.1007/s00468-014-1045-7

[B37] Kutschera U. Niklas K. J. (2013). Cell division and turgor-driven stem elongation in juvenile plants: a synthesis. Plant Sci. 207, 45–56. 10.1016/j.plantsci.2013.02.004 23602098

[B38] Lorimer C. Dahir S. Singer M. (1999). Frequency of partial and missing rings in Acer saccharum in relation to canopy position and growth rate. Plant Ecol. 143, 189–202. 10.1023/A:1009847819158

[B39] Loustau D. Berbigier P. Roumagnac P. Arruda-Pacheco C. David J. S. Ferreira M. I. (1996). Transpiration of a 64-year-old maritime pine stand in Portugal. Oecologia 107, 33–42. 10.1007/BF00582232 28307189

[B40] Masiokas M. Villalba R. (2004). Climatic significance of intra-annual bands in the wood of *Nothofagus pumilio* in southern Patagonia. Trees 18, 696–704. 10.1007/s00468-004-0355-6

[B41] Nabais C. Campelo F. Vieira J. Cherubini P. (2014). Climatic signals of tree-ring width and intra-annual density fluctuations in *Pinus pinaster* and *Pinus pinea* along a latitudinal gradient in Portugal. Forestry 87, 598–605. 10.1093/forestry/cpu021

[B43] Novak K. De Luis M. Gricar J. Prislan P. Merela M. Smith K. (2016). Missing and dark rings associated with drought in *Pinus halepensis*. IAWA J. 37, 260–274.

[B42] Novak K. de Luis M. Raventós J. Cufar K. (2013b). Climatic signals in tree-ring widths and wood structure of *Pinus halepensis* in contrasted environmental conditions. Trees 27, 927–934. 10.1007/s00468-013-0845-5

[B44] Novak K. Saz-Sánchez M. A. Cufar K. Raventós J. de Luis M. (2013a). Age, climate and intra-annual density fluctuations in *Pinus halepensis* in Spain. IAWA J. 34, 459–474. 10.1163/22941932-00000037

[B45] Olano J. M. Eugenio M. García-Cervigón A. I. Folch M. Rozas V. (2012). Quantitative tracheid anatomy reveals a complex environmental control of wood structure in continental Mediterranean climate. Int. J. Plant Sci. 173, 137–149. 10.1086/663165

[B46] Olano J. M. García-Cervigón A. I. Arzac A. Rozas V. (2015). Intra-annual wood density fluctuations and tree-ring width patterns are sex- and site-dependent in the dioecious conifer *Juniperus thurifera* L. Trees 29, 1341–1353. 10.1007/s00468-015-1212-5

[B47] Oribe Y. Funada R. Kubo T. (2003). Relationships between cambial activity, cell differentiation and the localization of starch in storage tissues around the cambium in locally heated stems of *Abies sachalinensis* (Schmidt) masters. Trees 17, 185–192. 10.1007/s00468-002-0231-1

[B48] Pittermann J. Sperry J. S. Wheeler J. K. Hacke U. G. Sikkema E. H. (2006). Mechanical reinforcement of tracheids compromises the hydraulic efficiency of conifer xylem. Plant Cell Environ. 29, 1618–1628. 10.1111/j.1365-3040.2006.01539.x 16898022

[B49] Rigling A. Bräker O. Schneiter G. Schweingruber F. (2002). Intra-annual tree-ring parameters indicating differences in drought stress of *Pinus sylvestris* forests within the Erico-Pinion in the Valais (Switzerland). Plant Ecol. 163, 105–121. 10.1023/A:1020355407821

[B50] Rigling A. Waldner P. O. Forster T. Bräker O. U. Pouttu A. (2001). Ecological interpretation of tree-ring width and intraannual density fluctuations in *Pinus sylvestris* on dry sites in the central Alps and Siberia. Can. J. For. Res. 31, 18–31. 10.1139/x00-126

[B51] Rossi S. Deslauriers A. Grièar J. Seo J.-W. Rathgeber C. B. K. Anfodillo T. (2008). Critical temperatures for xylogenesis in conifers of cold climates. Glob. Ecol. Biogeogr. 17, 696–707. 10.1111/j.1466-8238.2008.00417.x

[B52] Rozas V. García-González I. Zas R. (2011). Climatic control of intra-annual wood density fluctuations of *Pinus pinaster* in NW Spain. Trees 25, 443–453. 10.1007/s00468-010-0519-5

[B53] Rozenberg P. Van Loo J. Hannrup B. Grabner M. (2002). Clonal variation of wood density record of cambium reaction to water deficit in *Picea abies* (L.) Karst. Ann. For. Sci. 59, 533–540 10.1051/forest:2002038

[B54] Tessier L. Guibal F. Schweingruber F. H. (1997). Research strategies in dendroecology and dendroclimatology in mountain environments. Clim. Change 36, 499–517.

[B55] Tyree M. T. Ewers F. W. (1991). The hydraulic architecture of trees and other woody-plants. New Phytol. 119, 345–360.

[B56] Uggla C. Magel E. Moritz T. Sundberg B. (2001). Function and dynamics of auxin and carbohydrates during earlywood/latewood transition in Scots pine. Plant Physiol. 125, 2029–2039. 10.1104/pp.125.4.2029 11299382 PMC88858

[B57] Vaganov E. A. Schulze E. D. Skomarkova M. V. Knohl A. Brand W. A. Roscher C. (2009). Intra-annual variability of anatomical structure and δ^13^C values within tree rings of spruce and pine in alpine, temperate and boreal Europe. Oecologia 161, 729–745. 10.1007/s00442-009-1421-y 19653008 PMC2744769

[B58] Vieira J. Campelo F. Nabais C. (2009). Age-dependent responses of tree-ring growth and intra annual density fluctuations of *Pinus pinaster* to Mediterranean climate. Trees 23, 257–265. 10.1007/s00468-008-0273-0

[B59] Vieira J. Campelo F. Nabais C. (2010). Intra-annual density fluctuations of *Pinus pinaster* are a record of climatic changes in the western Mediterranean region. Can. J. For. Res. 40, 1567–1575. 10.1139/X10-096

[B60] Vieira J. Campelo F. Rossi S. Carvalho A. Freitas H. Nabais C. (2015). Adjustment capacity of maritime pine cambial activity in drought-prone environments. PLoS ONE 10:e0126223. 10.1371/journal.pone.0126223 25961843 PMC4427410

[B61] Vieira J. Rossi S. Campelo F. Nabais C. (2014b). Are neighboring trees in tune? Wood formation in *Pinus pinaster*. Eur. J. For. Res. 133, 41–50. 10.1007/s10342-013-0734-x

[B62] Vieira J. Rossi S. Campelo F. Freitas H. Nabais C. (2014a). Xylogenesis of *Pinus pinaster* under a Mediterranean climate. Ann. For. Sci. 71, 71–80. 10.1007/s13595-013-0341-5

[B63] Von Arx G. Carrer M. (2014). ROXAS – A new tool to build centuries-long tracheid-lumen chronologies in conifers. Dendrochronologia 32, 290–293. 10.1016/j.dendro.2013.12.001

[B64] Wilkinson S. Ogée J. Domec J.-C. Rayment M. Wingate L. (2015). Biophysical modelling of intra-ring variations in tracheid features and wood density of *Pinus pinaster* trees exposed to seasonal droughts. Tree Physiol. 35, 305–318. 10.1093/treephys/tpv010 25769337

[B65] Wimmer R. (2002). Wood anatomical features in tree-rings as indicators of environmental change. Dendrochronologia 20, 21–36. 10.1078/1125-7865-00005

[B66] Wimmer R. Strumia G. Holawe F. (2000). Use of false rings in Austrian pine to reconstruct early growing season precipitation. Can. J. For. Res. 30, 1691–1697. 10.1139/x00-095

[B67] Zalloni E. de Luis M. Campelo F. Novak K. De Micco V. di Filippo A. (2016). Climatic signals from intra-annual density fluctuation frequency in Mediterranean pines at a regional scale. Front. Plant Sci. 7:579 10.3389/fpls.2016.00579 PMC4852653 27200052

[B68] Ziaco E. Biondi F. Rossi S. Deslauriers A. (2014). Intra-annual wood anatomical features of high-elevation conifers in the Great Basin, USA. Dendrochronologia 32, 303–312. 10.1016/j.dendro.2014.07.006

