# Supplementary material for: Structure and Function of Intra–Annual Density Fluctuations: Mind the Gaps
Source: Front Plant Sci. 2016 May 6;7:595. doi: 10.3389/fpls.2016.00595 (PMC4858752; doi:10.3389/fpls.2016.00595)
Supplement: Supplementary file 2 [file DataSheet1.DOCX]

Supplementary Material

**Structure and Function of Intra–Annual Density Fluctuations: Mind the Gaps**

G. Battipaglia^1,2,3*^, F. Campelo^4^, Vieira J.^4^, M. Grabner^5^, V. De Micco^6^, C. Nabais^4^, P. Cherubini^7^, M. Carrer^8^, A. Bräuning^9^, K. Čufar^10^, , A. Di Filippo^11^, I. García-González^12^, M. Koprowski^13^, M. Klisz^14^, A. V. Kirdyanov^15,16^, N. Zafirov^17^, M. De Luis^18^

- **Correspondence:** Giovanna Battipaglia: [giovanna.battipaglia@unina2.it](mailto:giovanna.battipaglia@unina2.it)

Figure S1: Different types of Intra-Annual Density Fluctuations (IADFs) in *Pinus pinaster* from Portugal defined according to Campelo et al. (2007b): type E is a band of latewood-like tracheids within earlywood (in the first half of the ring); type L is a band of earlywood-like tracheids within latewood; type E^+^ is located between earlywood and latewood (gradual transition from earlywood to latewood); type L^+^ is located between latewood and earlywood of the successive tree ring (gradual transition from latewood to earlywood). Annual tree rings grew from left to right. no IADF= normal ring without IADFs.


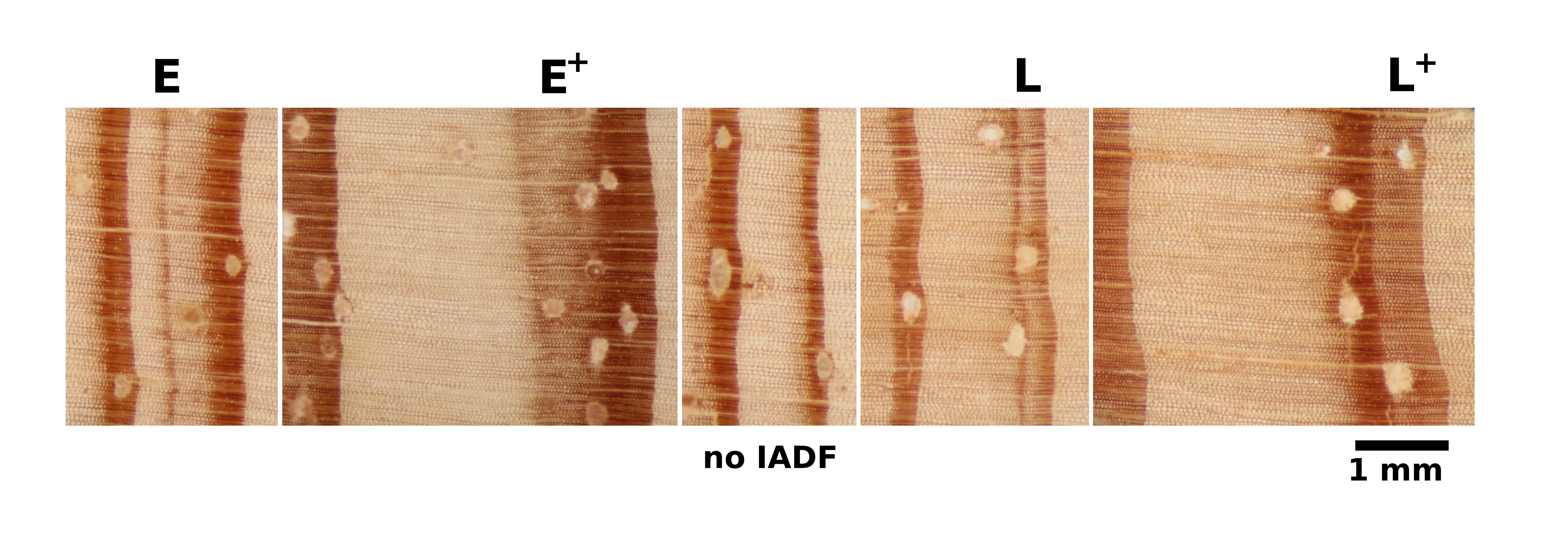
Photo modified from Campelo et al. (2013).


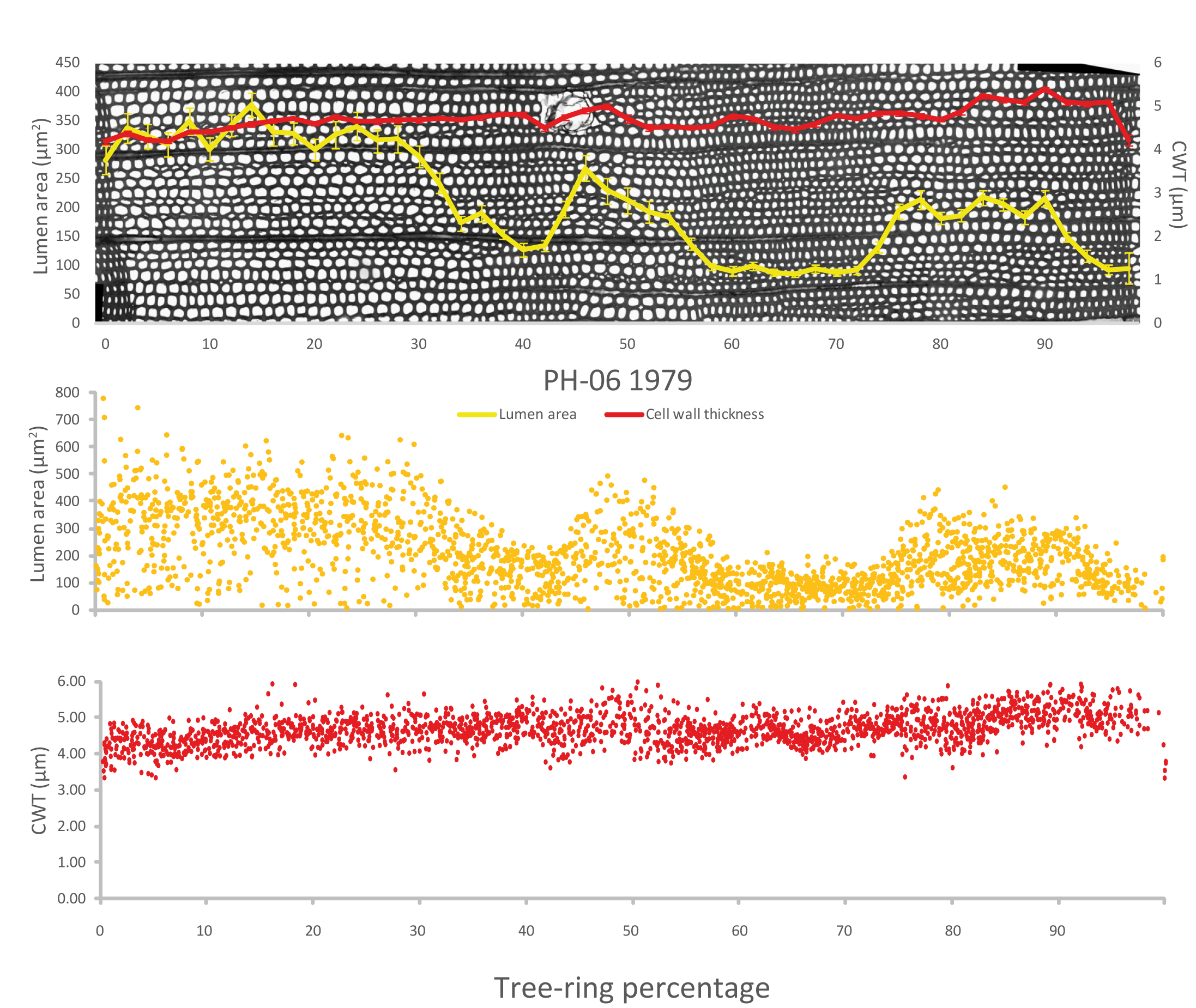
Figure S2: Example of quantitative wood anatomical analyses applied on Intra-Annual Density Fluctuations (IADFs). The upper image represents the year 1972 from a *Pinus halepensis* sampled near Zaragoza (Spain), where 13,772 cells were automatically recognized and lumen area (LD, *yellow*), cell-wall thickness (CWT, *red*) and relative position within the ring were collected for each one. The curves on the upper image were drawn splitting the ring into 50 equal sections and computing the mean (and SE) LD and CWT values for each section. The imprint of two IADFs with different duration and intensity can be observed. The middle and lower plots represent the raw values for LD and CWT, respectively.

# Supplementary: Stable isotopes approach

Five twin- cores of *Pinus pinea* from Italy, *Pinus halepensis* from Spain and Slovenia; *Pinus pinaster* from Portugal, *Larix decidua* from Poland and *Larix decidua* from Austria were used for stable isotope measurements. The tree rings were dated and occurrence and position of IADFs within them were recorded. In each core the IADFs related to the same year were split and the IADFs of the same type (E, E^+^,L, L^+^) were pooled together. The samples were ground with a centrifugal mill (ZM 1000, Retsch, Germany) using a mesh size of 0.5 mm to ensure homogeneity. Cellulose was extracted with a double-step digestion (Boettger et al., 2007; Battipaglia et al., 2008). The carbon and oxygen stable isotope compositions were measured at the CIRCE laboratory (**C**enter for **I**sotopic **R**esearch on the **C**ultural and **E**nvironmental heritage, Caserta, Italy). We report isotope values in the delta notation for carbon and oxygen, where δ^13^C or δ^18^O = (**R**_sample_ /**R**_standard_ – 1) × 1000, relative to the international standard, which is VPDB (Vienna Pee Dee Belemnite) for carbon and VSMOW (Vienna Standard Mean Ocean Water) for oxygen. R_sample_ and R_standard_ are the ^13^C/^12^C and ^18^O/^16^O ratios in the wood sample and in the standard. The standard deviation for the repeated analysis of an internal standard (commercial cellulose) was better than 0.1‰ for carbon and 0.2‰ for oxygen. Data of IADF were compared by means of ANOVA using SPSS statistical package (SPSS Inc., Chicago, Illinois, USA). Multiple comparison tests were performed with LSD, Bonferroni and Student–Newman–Keuls coefficients using **P** < 0.05 as the level of probability.

Table S1: IADF types distribution according to their position in the six analysed sites.

| **Type E** | **Type E^+^** | **Type L** | **Type L^+^** |
| --- | --- | --- | --- |
| Austria | Austria | Austria | Austria |
| Poland | Italy | Italy | Italy |
| Slovenia | Poland | Poland | Portugal |
|  | Portugal | Portugal | Spain |
|  |  | Slovenia |  |
|  |  | Spain |  |

Figure S3 : δ^13^C values (mean ± standard error) of different IADF types, across the different IADF sites, classified according to their position. Bars with different letters are significantly different (P < 0.05)


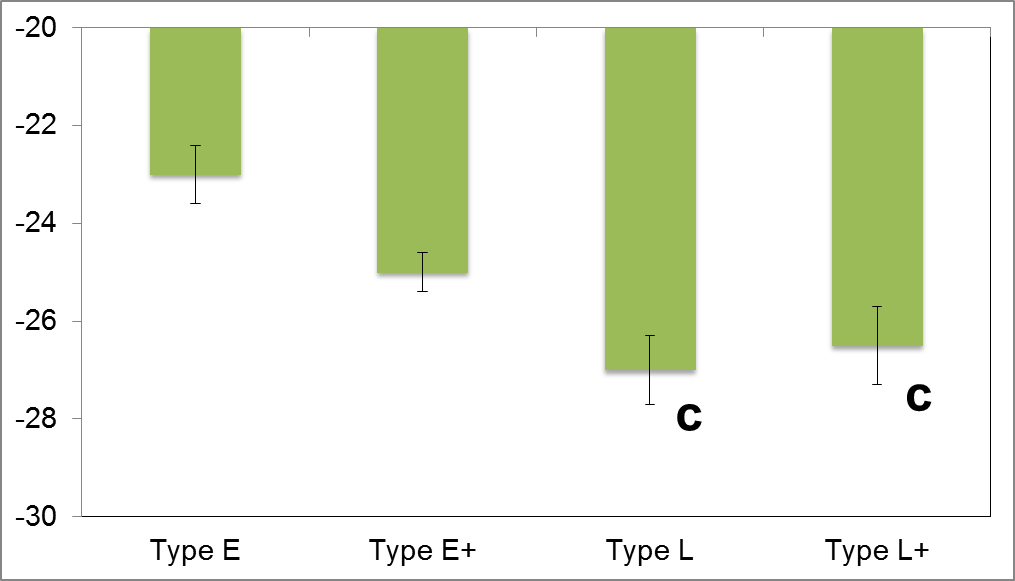


**a**

**b**

Figure S4: δ^18^O values (mean ± standard error) of different IADF types, across the different IADF sites, classified according to their position. Different letters indicate significantly different values (P < 0.05)


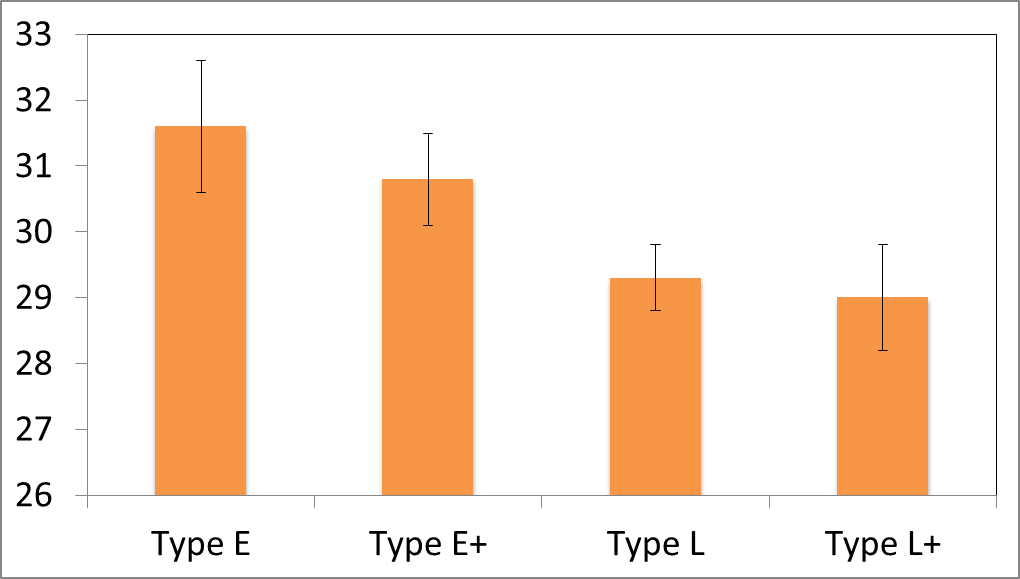


**a**

**a**

**b**

**b**

Table S1: Intra-Annual Density Fluctuations (IADFs) data catalogue. The compiled catalogue includes basic information of studied sites (coordinates, species), number of trees, samples and total tree-rings collected, and contact information of data owner. This catalogue is ”open” and we expect further data will be added in the future. The main objective of its presentation is to improve visibility of the available dataset to enhance collaborative research using large datasets. A full dataset including raw measurements and IADF identification has been also compiled, harmonized and is available at present for contributors for exploratory purposes. Agreement between data owners is however mandatory to develop any specific initiative.
